# Opportunities and challenges in stem cell therapy in cardiovascular diseases: Position standing in 2022

**DOI:** 10.1016/j.jsps.2022.06.017

**Published:** 2022-06-22

**Authors:** Shabnur Mahmud, Safaet Alam, Nazim Uddin Emon, Umme Habiba Boby, Kamruzzaman, Firoj Ahmed, A.S.M. Monjur-Al-Hossain, Afroza Tahamina, Sajib Rudra, Marzina Ajrin

**Affiliations:** a School of Health and Life Sciences, Department of Pharmaceutical Sciences, North South University, Dhaka 1229, Bangladesh; b Pharmaceutical Sciences Research Division, BCSIR Laboratories, Dhaka, Bangladesh Council of Scientific and Industrial Research (BCSIR), Dr. Qudrat-I-Khuda Road, Dhanmondi, Dhaka 1205, Bangladesh; c Department of Pharmacy, Faculty of Science and Engineering, International Islamic University Chittagong, Chittagong 4318, Bangladesh; d Department of Pharmaceutical Chemistry, Faculty of Pharmacy, University of Dhaka, Dhaka 1205, Bangladesh; e Department of Pharmaceutical Technology, Faculty of Pharmacy, University of Dhaka, Dhaka 1205, Bangladesh; f Department of Botany, Faculty of Biological Sciences, University of Chittagong, Chattogram 4331, Bangladesh; g Department of Pharmacy, University of Science and Technology Chittagong, Chittagong 4202, Bangladesh

**Keywords:** Stem cell therapy, Cardiac diseases, Bone marrow, iPSCs, MSCs, Pre-clinical trials, Clinical trials

## Abstract

This study intends to evaluate the development, importance, pre-clinical and clinical study evaluation of stem cell therapy for the treatment of cardiovascular disease. Cardiovascular disease is one of the main causes of fatality in the whole world. Though there are great progressions in the pharmacological and other interventional treatment options, heart diseases remain a common disorder that causes long-term warnings. Recent accession promotes the symptoms and slows down the adverse effects regarding cardiac remodelling. But they cannot locate the problems of immutable loss of cardiac tissues. In this case, stem cell treatment holds a promising challenge. Stem cells are the cells that are capable of differentiating into many cells according to their needs. So, it is assumed that these cells can distinguish into many cells and if these cells can be individualized into cardiac cells then they can be used to replace the damaged tissues of the heart. There is some abridgment in this therapy, none the less stem cell therapy remains a hopeful destination in the treatment of heart disease.

## 1 Introduction

Cardiovascular disease is a type of illness that includes the disorder of the heart or blood vessels in the body. It oversteps infectious disease and malignant fatal disease as the principal motive of death throughout the world (Taylor and Robertson, 2009). This disease is the foremost reason for fatality worldwide. Despite upliftment in pharmacological interventions, one in four women and one in three men face death within a year because of the myocardial infarction. In accordance with World Health Organization, around seventeen million people perish of cardiovascular disease every year, the greater number of them subsist strokes and heart attacks will need to sustain costly clinical treatment (Jadczyk et al., 2013). There are some recent treatment strategies such as regular exercise, proper diet, drugs and therapies, pacemakers, and revascularization therapy that have attained headway, these strategies may not be entirely efficient and do not recuperate permanently the task of the myocardium. Our body is a buildup of many different types of cells. Most cells are learned to redact particular functions; such as red blood cells carrying oxygen around the bodies in our blood, but they are incapable to divide. Stem cells give new cells to the body as it grows and replaced specialized cells that are damaged or lost. They have identical properties (e.g; They can split over and over again to generate new cells, after dividing, they can switch into the other types of cell that make up the body and they shows immunosuppressive capabilities) (Zhang et al., 2017). Stem cells are undifferentiated or partially differentiated in nature. But they have the strength to produce undifferentiated cells. And they can multiply to produce more and more stem cells. These cells are found in both embryonic or primary and adult organ systems and both of them have different characteristics. They are generally discriminated from the progenitor cells. Progenitor cells are cells that cannot divide indefinitely. There are several types of stem cells are available; for example, embryonic stem cells, bone marrow-derived stem cells, mesenchymal stem cells, pluripotent stem cells, cardiac stem cells, etc. Each of the cells has both advantages and drawbacks. For example, in the case of embryonic stem cells, they can individualize into specific cardiac cells but the drawback is that their use is unethical. Another is the mesenchymal cells, they have the convenience that they can be obtained from the patient’s own cells as they have an immunosuppressive property and they can easily be obtained from the source (Faiella and Atoui, 2016).

Stem cell therapy has the calibre to deflect the traditional treatment of cardiovascular diseases by incrementing the reanimate of the injured myocardium. In the last two decennia, there are lots of pre-clinical and clinical trials that have been redacted to determine the safety and efficacy of the different cell types (Mueller et al., 2018). In the case of usual treatment options, the treatment options are used only for managing the disease, but in the case of stem cell therapy, the strategy is used to recapture the damaged cell of the myocardium (Tompkins et al., 2018). This therapy is a new outskirt in the campaign in opposition to cardiovascular disease (Jadczyk et al., 2013). The dynamic of the stem cells and the object of the heart failure problem opened a rapid development in the area of human heart regeneration (Joggerst and Hatzopoulos, 2009). Cell therapy strategies are monumental because of the easement of use and a better safety profile (Nguyen et al., 2016). Stem cell therapy is the novel front-line fighter in the war against cardiovascular disease. They flake exquisite research and detraction. These cell therapies are desirable options to reanimate the myocardium and flourish the cardiac function after the myocardial infarction. Though stem cell therapies are one of the greatest obtainments in the treatment of heart disease, there are some irrational foundations for proving the extensive variety of stem cells and methods of isolation and production. Therefore, Stem cell therapy is assumed to reduce the mortality rate from cardiovascular disorders by 80% in individuals with high level of inflammation and damaged hearts. When stem cells are transplanted, they aid in the healing procrss of damaged tissues by using healthy cells to stimulate generation of new functional tissues. Stem cell transplantation may be beneficial for people with heart disease (coronary artery disease, valvular heart disease, cardiomyopathy, congenital heart disease, myocardial infarction, ischemic stroke, heart failure) due to the paracrine impact. When regenerative stem cells are implanted into wounded tissue, they are expected to produce substances that promote in the regrowth of healthy stem cells, decrease inflammation, stimulate the formation of new blood vessels, and prevent cell death and hypertrophy (Sun et al., 2016).

This study aims to evaluate the development of stem cell therapy in the treatment of cardiovascular disease, evaluate clinical and pre-clinical studies of stem cell therapy for cardiovascular treatment, slow down the adverse effects regarding cardiac remodelling, locate the problems of immutable loss of cardiac tissues. The novelty of the review focuses on the recent trends and advancements in the field of stem cell therapy in the treatment of cardiovascular disorders along with bridging with the ongoing knowledge.

## 2 Materials and methods

Collecting information is a methodological process of gathering information analyzing the information and evaluating the results. In this present study, primary data collection cannot be obtained. For that, all the data are collected in secondary parameters such as; library search, internet search and document search. To summarize and compile the desired findings related to stem cell therapy on cardiac degeneration treatments. Web of Science, PubMed, ScienceDirect, Scopus, Wiley Online Library, and Google Scholar databases were used to perform a literature search. In addition, genuine sources such as WHO, USFDA, and ClinicalTrials.gov were used to verify clinical trial data and other important information. Stem cells, stem cell treatment, drug discovery using stem cells, cardiovascular therapy using stem cells, clinical trials, case studies, retrospective studies, cohort studies, and *meta*-analyses are some of the keywords that were utilized in the searches. It was excluded from this evaluation any incomplete clinical trials, *meta*-analyses, case studies, cohort studies, or retrospective data from any of the above-mentioned legitimate databases. After the titles and abstracts of 200 publications and reports were discarded because they did not meet the inclusion criteria and only 80 unique sources remained for the final analysis.

## 3 Importance of stem cell therapy

The wear and tear theory in our body means the effects of aging that are caused by dynamic damage to the cells and the body systems over time. Our body wears out due to use. Once they wear out, they can no longer function properly. Repair of cells and tissues of the body in the wear and tear theory ensues throughout life but it declines with aging. Because of this chronic illnesses; such as cardiovascular symptoms are developed with age (Segers and Lee, 2008). This reparation action illustrates an ongoing symmetry between injury of the tissue and the reparative mechanism which is intermediated by subtle interaction between the anti-inflammatory signal and endogenous progenitor cells. At the former ages, risk factors are typically lower, as a result, there are enough cells are available to repair the small damage or injured cells. But at later ages, the repair cells decrease in number and in function. They cannot fulfill the needs of the reparative mechanism. In this case, cell therapy is an unprecedented approach that is made schematic to mitigate the failure of the endogenous repair (Taylor and Robertson, 2009).

## 4 Stem cells used in cardiovascular therapy and their origins

### 4.1 Embryonic stem cells and induced pluripotent stem cells

Embryonic stem cells can be obtained from the inner mass of the blastocyst and extended practically *in-vitro*. They can be able to uprising somatic cell lineages (Reubinoff et al., 2000). These stem cells can be pervaded into the corresponding organs. In pre-clinical results using embryonic stem cells, these cells showed a hopeful result. But till now, there was no clinical trials are conducted. There are some reasons behind this; such as using embryonic cells is non-ethical. Another reason these cells can differentiate into a cell that contains three germ layers such as ectoderm, mesoderm and endoderm (Poh et al., 2014). As a result, they occupy the ability to individualize any of the cell lineages. This causes the formation of teratoma at the administration site. The immunity problem is also a concern in using embryonic stem cells. As a result, the graft may be rejected (Lu et al., 2009). Aside from the pluripotent cells, ESCs can develop into any kind of cell, making them a gold standard for evaluating all other pluripotent cells grown *in vitro* (Lin et al., 2013). However, the use of ESCs in scientific investigations and therapeutic trials involving humans is limited owing to ethical constraints. To this purpose, iPSCs derived from adult somatic cells (e.g. skin fibroblasts) may be used to substitute ESCs since the process of generating iPSCs does not include the death of embryos (Takahashi and Yamanaka, 2006). iPSCs are comparable to ESCs, but they do not have the immunogenic or ethical constraints of ESCs, therefore they may be more therapeutically useful in the long term than ESCs are (Zhang et al., 2017). Furthermore, PSCs (pluripotent stem cells) may differentiate into cardiomyocytes for cardiac repair, although their therapeutic use is limited. They confront various obstacles, including the possibility of post-transplant rejection. Despite having immunological privilege, human amniotic fluid-derived stem cells (hAFSCs) do not differentiate into cardiac cells. Fang and colleagues used a Wnt modulating differentiation method to differentiate hAFSC-iPSCs into cardiac cells. hAFSC-iPSCs produced as a consequence. hAFSC-iPSC-CMs exhibit cardiac-specific markers that possess cardiomyocyte-specific electrical characteristics, and spontaneously contract. Importantly, the *in vitro* and *in vivo* findings indicate that these hAFSC-iPSC-CMs are low immunogenic, and overall, hAFSC-iPSCs might be promising candidates for heart regeneration cell therapy (Fang et al., 2020).

### 4.2 Skeletal myoblast

These are the first cell that undergoes both the pre-clinical and clinical trials. They are attained from the satellite cells, which is progenitor cell residing underneath the basal lamina of the skeletal muscle fibers. These cells can be easily obtained from the sources by the muscle biopsies, they can be easily extended into the outside media, they are resistant to the ischemic condition and they have a low risk of tumour formation (Hassan et al., 2014). Because of this reason, these cells were motivated for use in cardiovascular disease. This cell has the ability to individualize into myotubes, they have declined myocardial fibrosis, and remission of the ventricular remodelling and also, they show an improvement in the low ventricular ejection fraction. Despite these advantages, these cells are encouraged to ignore because they tend to cause arrhythmia. In some studies, most of the patients suffered from arrhythmia after the administration of the skeletal myoblast cells. Studies suggested that this arrhythmia is caused by the lacking of the skeletal cell to conduct electromechanical coupling with the cardiomyocytes (Almeida et al., 2015). Another study, which was the large randomized, placebo-controlled and double-blinded trial, suggested that when the skeletal myoblast cells were given to the patient intramyocardially, the patient’s condition did not improve after a 6-month follow-up. And a large number of people had suffered from abnormal heart rhythm (Brickwedel et al., 2014). Because of these drawbacks, the focus on the use of skeletal myoblast has declined.

### 4.3 Bone marrow (BM) derived stem cells

BM illustrates immature and heterogeneous cell demography. Whenever there causes an injury, bone marrow-derived cells are required to regenerate the damaged tissues. According to a murine model study conducted in 2001, murine bone marrow-derived cells when administered intramyocardially, they promote cardiac function in the murine model of myocardial infarction (Orlic et al., 2001). Using these cells is a less ethical concern and these cells can be easily isolated from the source and they can be self-renewed. A large number of autologous cells can be obtained from bone marrow-derived cells (Estronca and Ferreira, 2016).

### 4.4 Bone marrow (BM) derived mononuclear cells

BM-derived mononuclear cells are also heterogeneous cells. It includes different types of small cell populations; such as hematopoietic stem cells, mesenchymal stem cells, and endothelial progenitor cells. Among them, hematopoietic stem cells are occupied a major portion (Dimmeler and Zeiher, 2009). According to the trial conducted in 2001, bone marrow-derived mononuclear autologous cells are injected intracoronary route through density gradient centrifugation six days after myocardial infarction. Results showed an importantly declined in the infarct size and promote contractility and perfusion. A large number of controlled, randomized trials exhibited an improved cardiac function after the administration of bone marrow-derived mononuclear cells via intramyocardially and intracoronary. But many studies also failed to demonstrate the efficacy of these cell effects (Lunde et al., 2006).

### 4.5 Bone marrow (BM) derived hematopoietic stem cells (HSCs) and endogenous progenitor cells (EPCs)

Mature hematopoietic stem cells are multipotent stem cells they are located in the bone marrow and they can generate different types of blood cells. Endogenous progenitor cells illustrate a pro-vasculogenic subset of hematopoietic stem cells in the bone marrow. They present a population of surface markers such as CD34 and CD133. According to a phase 1 clinical study in 2001, autologous bone marrow CD133+ cells was injected in the infarcted area at the time of coronary artery bypass grafting in six patients. After three months, four patients presented an improved global left ventricular ejection fraction and five patients presented increased infarcted tissue perfusion. In accordance with the study, it is assumed that intra-myocardial administration of bone marrow-derived CD133+ cells are immune and incite angiogenesis (Nasseri et al., 2014). A later phase 2 trial ensured that these cell uses improved the left ventricular ejection fraction and myocardial perfusion after six months of administration of the stem cell transplantation as compared to the standard group. Despite these results, a long-term follow-up study plucked to present an improved cardiac function in the stem cell treated patients after several years (Nasseri et al., 2014). In the former phase 2 and 3 trials, the scar size and the perfusion rate were importantly increased after the myocardial transplantation of CD133+ stem cells. Nevertheless, there were no effects on the global function were detected from the study. Another phase 3 trial; known as PERFECT, NCT00950274, showed a reduced scar size and improvement of the myocardial perfusion, but there were no significant differences in the left ventricular ejection fraction was unearthed after the administration of the CD133+ stem cells. The study was a randomized, placebo-controlled and double-blinded study (Donndorf et al., 2012).

### 4.6 Bone marrow (BM) derived mesenchymal stem cells (MSCs)

Bone marrow-derived mesenchymal stem cells are the sub-population stem cells that are multipotent (Adibkia et al., 2021). They are the non-hemopoietic stem cells under plastic-adherent situations. These cells can be individualized into osteoblasts, and adipocytes under an *in-vitro* environment and they express the cell markers such as CD73, CD105, CD90. But they have to lack to express some cell markers; such as CD34, CD45, HLA-DR (Dominici et al., 2006, Adibkia et al., 2021). A current *meta*-analysis, which includes 58 pre-clinical trials, reported that total infarct size is reduced by about 7% and improved cardiac function by 11% which is occurred after the administration of the mesenchymal cells in the animal models of acute myocardial infarction and ischemic myopathy (Jeong et al., 2018). Another study conducted by karantalis et al. exhibited that patients with coronary artery bypass surgery were injected with mesenchymal stem cells intramyocardially resulting in a decreased scar size and improvement of the tissue perfusion and the increasing of the regional function (Karantalis and Hare, 2015).

### 4.7 Cardiac stem cells and progenitor cells

The adult human heart is deliberated as a post-mitotic organ traditionally that has the capacity for self-renewal. In the mature heart, a self-renewal c-kit+ cell is first discovered by Beltrami et al. this c-kit+ cell can be individualized into cardiomyocytes cells, smooth muscle cells and endothelial cells (Beltrami et al., 2003). These cells support the reanimation of the injured tissues (Fathi et al., 2020). Over the past decade, there are diverse cardiac stem cells and cardiac progenitor cells are established; such as cardio-sphere derived cells, stem cell antigen cells etc. Different types of studies demonstrated that cardiac stem progenitor cells can avail to cardiomyogenesis by straight differentiation. Different in-vitro experiments showed a promising result using cardio derived cells. A systemic trial that included 80 animals stated that after the administration of cardiac stem cells to the animals, the left ventricular ejection fraction increased by 11% as compared with the placebo groups. The first phase 1 clinical trial, named SCIPO, NCT00474461 conducted in 2011 (Magenta et al., 2013), evaluated that after the administration of c-kit+ cardiac stem cells by intracoronary route to the patients with ischemic cardiomyopathy, there observed no adverse effects and showed reduced mortality. Another trial, named CADUCEUS (NCT00893360) confirmed the advantages of cardiac stem cell therapy. In this study, cardiac stem cells were grown from the endomyocardial biopsy tissues and delivered to the patients who were suffering from left ventricular dysfunction following myocardial infarction (Malliaras et al., 2014). There was another study conducted, named PERSEUS (NCT01829750) phase 2 randomized controlled clinical trial, which showed that cardiac stem cells treatment alternates the left ventricular function compared to the control group three months later (Ishigami et al., 2017).

### 4.8 Cardiac c-kit+ stem/progenitor cells

The first stem cells discovered in putative cardiac stem cells (CSCs) were cardiac c-kit+ cells, which have been shown to play a crucial role in CSCs proliferation. According to recent research, c-kit+ cells may aid in the regeneration and repair of the heart (Fathi et al., 2020). Cardiac stem and progenitor cells include c-kit receptor, sca-1, cardiosphere-derived cells (CDCs), islet-1+ cells, and epicardial-derived cells (EPDCs) (Nasser et al., 2020). C-kit+ cells have a stronger capacity for development than other CSCs, and they have been used as a marker to identify CSCs (Zhou and Wu, 2018). A heart attack in a mutant animal with deficient c-kit signaling results in worse cardiac remodeling, while transgenic mice that overexpress c-kit in the heart have enhanced myocardial recovery. According to Beltrami et al, cardiovascular c-kit+ cells are multipotent, self-renewing, clonogenic and capable of producing vascular smooth muscle cells (VSMCs), cardiomyocytes (CMCs), epithelial cells and vascular smooth muscle cells (VSMCs) (Senyo et al., 2013). Recent studies indicate that C-Kit is an essential receptor for several stem cell types, including germ cell precursors, hematopoietic precursors, and CSCs (Sun et al., 2016). Differentiated or undifferentiated C-kit+ CPCs may contribute to the advancement of cell-based treatment approaches. Treatments based on C-kit+ CPCs might potentially benefit from more research into optimum treatment timing, cell delivery methods, and cell angiogenesis (Fathi et al., 2020).

### 4.9 Induced pluripotent stem cell for the cardiac regeneration

Cardiac regeneration is an important objective in regenerative medicine because of the growth of heart failure. In human stem cell biology, iPSCs may be used to create tissue-specific cells that are similar to those created from a human embryo, but without having to harm the embryo. Injecting iCM into the hearts of mice and pigs with models of heart failure (MI) has been investigated in many research (Youssef et al., 2016). iPSC-derived cells were used to treat an acute MI mouse model by Nelson et al. Four weeks following heart surgery, the iPSC-injected animals showed better left ventricle function and regional wall motion than the fibroblast-injected animals, according to echocardiography. Subcutaneous and intramyocardial injections of iPSC resulted in teratomas in immunocompromised mice. There were no teratomas in immunocompetent mice, as previously stated (Ge et al., 2012). Teratomas have been found in immunocompetent and SCID mice after they were injected with undifferentiated iPSCs inside their hearts. A study by Kim et al; about how iPSCs grew into the body of SCID mice. After four weeks, the iPSCs had grown into the body. In spite of the stated increase in myocardial function as well as myocardial vitality, they found evidence of teratoma development (Kim et al., 2015). The study shown that cytokines secreted by iPSCs may play a mechanistic role in the restoration of heart function, even if teratomas occur. In a rat model of MI, undifferentiated iPSCs had similar effects as undifferentiated murine ESC, which resulted in teratoma development in the early experiments (Nussbaum et al., 2007, Zhang et al., 2011). Therefore, the summury of the origin of stem cells for the therapies od cardiac diseases have been illustrated in Table 1.

**Table 1:** Stem cells used for cardiac diseases and their origins.

Type of stem cell	Source	Advantages	Disadvantages	References
Embryonic stem cells	Inside the cell mass of the pre-implantation blastocyst.	Limitless self-renewal retention; pluripotency is observed and limitless proliferation.	Prohibited for ethical concern, can cause teratoma.	(Bilic and Belmonte, 2012, Parish and Thompson, 2021)
Bone marrow-derived (mononuclear, HSCs, EPCs, MSCs) stem cells	Located in the bone marrow, umbilical cord, placenta, peripheral blood, they are obtained from bone marrow, adipose tissues.	Easier to separate, safe and practicable to the implant, they are easy to separate, dilate in the culture, lower immunogenic effects, Multipotent.	There is a suspect of obtaining the true cardiac cell from the bone marrow-derived cells. they are largely heterogenic, they have heterogenic differentiation.	(Ratajczak et al., 2007, Guo et al., 2020)
Endothelial progenitor cells	Peripheral blood, bone marrow	Easily put in motion and present in the peripheral blood, they are necessary for neovasculogenesis.	They are little in populations, heterogenic property, in the cardiovascular mobility, their number decreases.	(Urbich and Dimmeler, 2004, Kolesnichenko et al., 2021)
Skeletal myoblast	Located in the advanced muscle between the basement membrane and the sarcolemma.	They are resistant to ischemia, multipotent, they do not cause teratoma.	Dynamic for the arrhythmia, they can less differentiate into cardiomyocytes.	(Araki et al., 2021, Jalal et al., 2021)
Cardiac stem cells	Specific nook in the myocardium.	They are resident cells, they have strong cardiovascular differentiation, no teratoma formation.	Difficult to obtain.	(Messina et al., 2004, Bearzi et al., 2007)

## 5 Stem cell-mediated improvement of heart function

According to Nguyen et al., it is raised that stem cell therapy are assumed to perform by adult stem cells can yield new cardiac cells or tissue by the process called angiogenesis. But later inquiry disclosed that a stem cell can be inculcated into the damaged myocardium by differentiating into cardiomyocytes (Nguyen et al., 2016, Quaife-Ryan, 2020). Another mechanism was that the stem cells arouse the endogenous progenitor cells and originate vasculature by the process of vasculogenesis or angiogenesis. But this mechanism remains argumentative because of the scarcity of the surface markers to recognize the stem cells. Only a set of endogenous progenitor cells are capable of producing neovasculogenesis. Their amount is very low to improve heart function (Nguyen et al., 2016). The next mechanism is the paracrine effects of the stem cells. Stem cells obtained from the adult source assert the paracrine effects by releasing the factors which are cardioprotective. By the activation of this pathway, vascular remodelling and reperfusion injury can be stingless in patients who are suffering from heart failure and acute myocardial infarction (Jiang et al., 2017).

## 6 Use of mesenchymal cells in heart diseases

Mesenchymal stem cells are the most broadly used stem cells that are nowadays being tested for the heart. There are some reasons behind this; such as, easily attainable in the tissues, such as bone marrow tissues, fat tissues and others. They can largely expand in the ex-vivo. They have also immunosuppressive properties. Mesenchymal stem cells decline the scar size and enhance the left ventricular ejection fraction after implantation in most of the animal models. They also increase vascular density and myocardial perfusion (Tang et al., 2004). According to a current phase 1 clinical trial which was a randomized, double-blinded and placebo-controlled dose-escalation study, single dose admiration of the allogenic mesenchymal cells with acute myocardial infarction was recorded to be out of danger. Mesenchymal cells also improve the outcomes of the cardiac arrhythmias, pulmonary functions, left ventricular functions etc. according to a hypothesis, mesenchymal cells can individualize into cardiomyocytes and corroboratory cell types to refit the cardiac tissues (Trachtenberg and Hare, 2010). But in accordance with some of the hypotheses, it is shown that most of the mesenchymal cells are entangled in the lungs and the capillary tissues rather than the heart. Mesenchymal cells release some growth factors and cytokines such as VEGF, MCP-1, HGF, FGF and thrombopoietin. These factors cause arteriogenesis and they clinch the stem cell crypt in the intestine. They protect in opposition to ischemic renal and limb tissues lesion. They also support to maintain hematopoiesis (Schraufstatter et al., 2011). Besides, relying on the method of administration, 6% or less of the mesenchymal cells that are transplanted can exist in the heart. Transplanted mesenchymal cells can individualize into the cardiomyocytes and the ventricular activity was quickly gathered in less than 72 h after transplantation. These factors also evaluated the favorable effects of the mesenchymal cells on the cardiac system which also includes neovascularization. Early clinical studies regarding the use of mesenchymal cells assessed that these cells enhanced the cardiac function and they have quick cardioprotective effects and these effects are due to the paracrine effects of the mesenchymal cells rather than the replacement effect (Lai et al., 2011).

## 7 Paracrine effects of mesenchymal cells

Mesenchymal cells secreted a large number of the growth factors and cytokines which assert paracrine effects (Caplan and Dennis, 2006). A study conducted by Gnecchi et al. demonstrated that secretion from the cell can singly enhance the cardiac function in the animal model of acute myocardial infarction (Gnecchi et al., 2008). Mesenchymal cells which are obtained from the embryonic stem cells or fetal tissues and cultured can decline the scar size in the animal models of myocardial ischemia. The exosome is the elementary moderator of the mesenchymal cell’s paracrine effects (Lai et al., 2011). Trials based on cell doses and route of administration have been mentioned in Table 2.

**Table 2:** Trials based on cell doses and route of administration.

Serial Number	Study nature	Results	References
1	Allogenic mesenchymal cells are used in the swine in three different doses (1,3,10 million) after a 75 min left anterior descending coronary artery repression.	The former dose (3 and 10 million) showed a significant increase in the left ventricular systolic function and pre-load recruit stroke work in comparison with the control group.	(Golpanian et al., 2016)
2	Allogenic mesenchymal cells were administered intramyocardially at the infract border zone in four doses (25, 75, 225, 450 million), one hour after experimental acute myocardial infarction.	Lower cell doses (25 and 75 million) decreased the infarct expansion and remodelling in comparison with the control group. Reduction in the left ventricular end-diastolic volume and end-systolic volume.	(Hamamoto et al., 2009)
3	Stem cells were delivered via direct injection in the open chest pigs.	High dose autologous mesenchymal cells (200 million) reduced the infarct size in comparison with the low dose (20 million) in the post-acute myocardial infarcted swine. Improvement of contractility was observed in both groups.	(Lee et al., 2011)
4	Administration of mesenchymal cells through endomyocardial route.	A significant reduction in the infarct size was observed in the lower dose group (24 and 240 million). The higher dose (440 million) did not show any improvement.	(Hashemi et al., 2008)
5	Three clinical trials were conducted to assess the relationship between the cell dose and clinical efficacy. Autologous CD34+ cells were administered via the intracoronary route into the infarcted artery after eight days of stenting and three different doses were used (5,10,15 million).	Significant improvement in perfusion was observed in the group treated with 10 million CD34+ cells. Improvement in the left ventricular ejection fraction was observed in the patient treated with 5 million stem cells as compared with the control.	(Poole and Quyyumi, 2013)
6	Allogenic mesenchymal cells were administered via the intracoronary route and trans endocardial route in a canine model with acute myocardial infarction.	Cells administered via trans endocardial route increased left ventricular ejection fraction, left ventricular end-diastolic volume, left ventricular end-systolic volume. There was no change observed in the cells administered via intracoronary route. Trans endocardial injection decreased myocardial ischemia.	(Perin et al., 2008)
7	Administration of the adipose-derived stem cells in a porcine model of acute myocardial infarction to compare the intracoronary and trans endocardial route.	Neovascularization was increased in the cell therapy given by the intracoronary route compared with the trans endocardial. Both delivery mechanisms evolved at the same rates of engraftment.	(Rigol et al., 2010)

## 8 Autologous CD34^+^ stem/progenitor cell therapy

CD34 is the cell surface marker that is primarily manifest in the endothelial cells, hematopoietic stem cells and vascular endothelial progenitor cells. CD34^+^ markers on endothelial progenitor cells are placed in the peripheral blood and in the bone marrow. When inflammation signals occur in the vascular system or ischemia occurs, these cells put in motion from the bone marrow, migrate and are centered in the sites and cause angiogenesis and vasculogenesis. By this, they can contribute to mature vascularization. Patients sustained with radiation and chemotherapy need CD34+ hematopoietic stem cells to regenerate and restore the hematopoietic system in the body. In animal studies, CD34+ cells cause angiogenesis in the case of ischemia. Several studies presented the safety and efficacy of the CD34+ cells in the treatment of cardiac disease. Currently, a phase 3 trial has been started to discover the role of CD34+ cells in myocardial and peripheral ischemia. These cells are proved safe and better tolerated in the treatment of acute myocardial infarction, heart failure and angina models. Various studies explored that CD34+ cells have a benefit in patients with coronary microvascular disease. There are several investigations over the last decade, which conclude encouraging data on the use of CD34+ cells in the treatment of cardiac disease; such as acute myocardial infarction, dilated cardiomyopathy and heart failure. There are some limitations also, including a limited number of patients, highly selected patients, lack of randomized trials. Because of this, a large number of randomized and blinded studies are needed to assess the efficacy of the cells, their delivery routes, and their isolation process. The available studies have many drawbacks and must be interpreted with precaution while continued examination evaluating the role of autologous CD34+ cells in larger well-designed studies are undertaken. CD34+ cells may probably be a potential treatment modality for patients who have limited options such as coronary endothelial and microvascular dysfunction. So further studies are essential to understand the dynamic role of CD34+ cells in the treatment of symptomatic populations who have non-obstructive coronary artery disease, microvascular disease, and endothelial functional problems (Prasad et al., 2020). The Autologous CD34+ stem/progenitor cell therapy. The state-of-the-art of CD34+ cell therapy and its clinical trials for cardiovascular diseases have been presented in Table 3.

**Table 3:** Summary of the state-of-the-art of CD34+ cell therapy and it’s clinical trials for the cardiovascular diseases (Prasad et al., 2020).

Authors	Country	Sample size	Disease	Design	Delivery	Follow-up	Results
Quyyumi et al;	USA	168	STEMI	Randomized double-blinded	Intra-coronary	6 months	Reduced infarct size, reduced MACE, improved ejection fraction
Losordo et al;	USA	167	Refractory angina	Randomized double-blinded	Intra-myocardial	1 year	An important decline in the angina frequency. Improvement in the work-out tolerance.
Wang et al;	China	112	Refractory angina	Randomized double blinded	Intra-coronary	6 months	Significant reduction in angina frequency and enhancement in myocardial perfusion.
Lee et al;	Taiwan	38	Refractory angina	Randomized double-blinded	Intra-coronary	1 year	Decreased angina, improved ejection fraction.
Vrtovec et al;	Slovenia	110	Dilated cardiomyopathy	Randomized double-blinded	Intra-coronary	5 years	Increased LVEF, Increased 6MWD and decrease in NT pro-BNP, decreased mortality.
Bervar et al;	Slovenia	38	Dilated cardiomyopathy	Randomized double-blinded	Trans-endocardia	1 year	Increased 6MWD, decrease in NT-pro BNP, and significant improvement in diastolic parameters
Lezaic et al;	Slovenia	21	Dilated cardiomyopathy	Randomized double-blinded	Intra-coronary	6 months	Improved rest myocardial perfusion, LVEF, 6-minute walking distance.

## 9 Pre-Clinical and clinical trials on stem cell therapy in case of cardiovascular diseases

### 9.1 Pre-Clinical trials on stem cell therapy in case of cardiovascular diseases

Preclinical trials are a very nifty tool to go forward to the clinical trial phase and can give hints about prospective outcomes so that researchers and other associates can assure probable preparations before the clinical trials (Yan et al., 2020). A suitable animal model that accurately reflects human pathological conditions is required for preclinical development. Prospective medical interventions for heart disease are typically tested first in small animal models (rodents), a model that allows for relatively quick and inexpensive testing, as well as large enough group, sizes to confirm statistical significance. The assessment of cardiovascular outcomes in rodents has been improved with the help of recent technological advances in PET-MRI (positron emission tomography/magnetic resonance) imaging and echocardiography (Santos et al., 2015). Again, cardiac repair studies demonstrated more pronounced actions in a rodent model by increasing left ventricular ejection fraction (LVEF) up to 20%, and normalizing LV function while in large animal studies the value of mean LVEF augmentation of 5 to 7% (Zwetsloot et al., 2015). This moderate benefit is more in line with clinical trial results, providing a more realistic picture of the expected benefit of human cell-based cardiac therapies. The presence or absence of collateral coronary circulation is a critical consideration when selecting an animal model for a study. Many of these criteria are met by large animals such as pigs, dogs, or sheep. Dogs have a large collateral coronary circulation, whereas pigs and sheep, like humans, do not have a functionally relevant vascular adaptation process (MAXWELL et al., 1987). As a result, a dog model is well suited to studying vascular adaptation to myocardial ischemia, whereas pigs and sheep are commonly used to evaluate the direct myocardial impacts of hypoxic injuries (Yang et al., 2021). Notable pre-clinical trials on stem cell therapy in case of cardiovascular diseases have been represented in Table 4.

**Table 4:** Pre-Clinical trials conducted on stem cell therapies (Tompkins et al., 2018).

Types of stem cell	Small animal trials	Large animal trials
Bone marrow-derived mesenchymal cells	Small animal trials have presented an auspicious upshot after acute myocardial infarction. Reduction of the infarct size and also fibrosis. Onward of left ventricular ejection fraction and vasculogenesis. Excessive amenities includes-reduced apoptosis reduced fibrosis and improved vascular endothelial growth factor expression. Enhance the regional blood flow in the infarct zone.	In the swine, acute and chronic myocardial infarction model, not only the autologous but also allogeneic mesenchymal cells showed a better result. Improvement in left ventricular ejection fraction and reduction of scar size and also improvement thickness. In the no-infarcted region, contractility was increased.
Adipose-derived stem cells	Adipose-derived stem cells and bone marrow-derived mesenchymal cells are administered intramyocardially into the rats after one week of post-myocardial infarction. None of them improve angiogenesis or reduced the infarct size. But the adipocyte-derived cells increase left ventricular ejection fraction. CD29+ and CD29+ cell markers of the adipose tissues decreased the infarct size and promote the left ventricular function.	These cells were used in the rabbits which had chronic ischemia. After 3 weeks of myocardial infarction, the rabbits have administrated adipose-derived cells into the infracted myocardium. Results include- Eminent vascular density left ventricular ejection fraction, end-diastolic volume compared with the 5-week post-injection. Allogenic adipose-derived cells improved perfusion but they did not improve the left ventricular ejection fraction in the porcine model when given via intracoronary. After the administration of adipose-derived cells in humanized pigs, 4 weeks post-myocardial infarction, there was improved perfusion and decreased infarct size in higher concentrations. Lower concentration did not show any effects.
CSCs/Cardiac progenitor cells	Cardiac c-kit cells are capable of self-renewal and they operate in a multipotent and clonogenic system to yield cardiomyocytes, smooth muscle cells and endothelial cells. They show more engraftment and individualization. better improvement in remodelling and reduced scar size compared to mesenchymal. They acted thirty-fold better than MSCs.	In a chronic ischemic swine model, intracoronary administration of c-kit+ CSCs into pigs 3 months post-MI demonstrated the therapeutic efficacy of these cells. Beginning 1-month post-injection, the LVEF rose in the cell-treated group and there was a regional increase in cardiac function. CSCs engrafted and some differentiated into cardiomyocytes and vascular structures.
Cardio spheres and cardio sphere-derived cells	Allogenic cardio derived cells were injected into rats by the intracoronary route and resulted in a decreased scar size and improved cardiac function, myocyte cycling and angiogenesis. Allogenic cardiac cells are proved efficient in the revitalization of senescent rats.	Treatment with cardio derived cells showed a beneficial treatment in acute and chronic myocardial infarction. These effects are mediated by cardio derived exosomes.
Bone marrow-derived mononuclear cells	Bone marrow-derived mononuclear cells were administrated intramyocardially and the results represented that this cell therapy promotes vasculogenesis at two weeks post-injection. But in the four-week group, there did not show any increase in vascularity. It may have been secondary to the maturation of the scar.	Improvement of wall thickening occurred four weeks after myocardial infarction. There also noticed a rise in the vascularization of the myocardium and a decline in the scar size. Bone marrow-derived mononuclear cells showed a variable result in the ventricular function in large animal models.
Pluripotent Stem Cells	Administration of the pluripotent stem cells in the myocardium of the mice via direct injection results in increased heart function and engraftment. Wall thickness was increased and declined fibrosis. But the long-term benefit of using pluripotent stem cells is not properly studied.	In the large animal study, the combination of pluripotent stem cells and human mesenchymal cells were used in swine. The combination of the cells increased vasculogenesis. But these therapies increase capillary density and sometimes caused apoptosis.

### 9.2 Clinical trials on stem cell therapy in case of cardiovascular diseases

#### 9.2.1 REPAIR-AMI trial

It is a double-blinded, placebo-controlled, multicentered trial design. Here 204 patients were used who had re-perfused acute myocardial infarction to receive bone marrow-derived progenitor cells via intracoronary route into the infarct artery 3 to 7 days after infarct reperfusion therapy. From this statistical analysis of the REPAIR-AMI trial, it is shown that MRI analysis of left ventricular function was performed after 24 months and assessed that the left ventricular ejection fraction between placebo and BMC group was statistically significant after adjustment from baseline derived from quantitative LV angiography (p = 0.009). left ventricular volume did not present any significant differences between the placebo and BMC group, though there was s decrease in LV systolic volume in the BMC group. There was a decline in the infarct size in the BMC group compared to the placebo group. The wall thickening of the infarcted segment was improved in the BMC group in comparison to the placebo group (Assmus et al., 2010).

#### 9.2.2 BOOST trial

The study aimed to determine whether a single intracoronary infusion of autologous bone marrow cells had an impact on left ventricular ejection fraction in the patient after ST elevated myocardial infarction. In this study, 60 patients were used the BMC was given to the patient via the percutaneous intracoronary route. There was an improvement of LVEF by 6% at 6 months and 2.8% was observed in the patient. From the study, it is concluded that single BMC administration did not promote a sustained improvement of LVEF in ST elevated myocardial infarction patients. After a six-month follow-up, there observed a significantly better recovery of LVEF when compared with the control group (p = 0.003). the difference declined at 18 months (p = 0.07) and there observed no differences at 61 months. From this study, it is concluded that BMC administration has no longer active in the patients (Meyer et al., 2009).

#### 9.2.3 POSEIDON trial

This trial was performed to assess the safety and efficacy of the allogeneic and autologous mesenchymal cells in the patients who had LV dysfunction due to ischemic cardiac myopathy. It was a randomized trial with 30 patients having LV dysfunction. allogenic mesenchymal cells reduced LV end-diastolic volume. Low dose concentration produced a greater decrease in the left ventricular volume and an increase in ejection fraction. But allogeneic mesenchymal cells did not stimulate a significant alloimmune response (Hare et al., 2012).

#### 9.2.4 CADUCEUS trial

The study aimed to determine the safety and efficacy in a randomized, controlled trial in a full one-year result. In this study, autologous cardiosphere derived cells were used which were obtained from endomyocardial biopsy and were given to the patient via the intracoronary route in 17 patients with left ventricular dysfunction. From the study, it was determined that CDC-treated patients had a smaller scar size compared to the control. CDC-treated patients had an improved regional function of the infarcted segment but the administration of CDC to the patients did not raise significant safety issues (Malliaras et al., 2014).

#### 9.2.5 TOPCARE-AMI trial

It was the first randomized study which aim was to determine the effects of intracoronary infusion of circulating CPC or bone marrow-derived cells in 59 patients. Five years of follow-up data were completed. Serum NT- pro-BNP significantly decreased at five years follow up which indicated the absence of heart failure. There was a persistent improvement in the left ventricle ejection fraction and a reduction in the infarct size was observed (Leistner et al., 2011).

#### 9.2.6 Trials on the combined stem cell therapy

The combination of cell therapy is a novel accession in improving therapeutic efficiency. Some large and small animal trials united the different cell therapy and progenitor cells. Combination of the cells associated with growth factors improves vasculogenesis, increased cell progress, declines apoptosis and improves cardiac function (Tompkins et al., 2018).

#### 9.2.7 Small animal studies

Ott et al. administered skeletal myoblast and bone marrow mesenchymal cells in the myocardium of the rats after 7 days of infarction. After 8 weeks, the combination community increased an ejection fraction, left ventricular ejection fraction and left ventricular end-diastolic volume and the retention of the bone marrow mesenchymal cells. When a similar combination was given to the animals two weeks post-infarction via intramyocardially, similar results were obtained from the study compared with the individual cell group (Ott et al., 2007). According to Quijada et al., a combination of the murine mesenchymal cells and cardiac progenitor cells was used which is termed cardiac chimaeras. This study was performed to evaluate the efficacy of these cardiac chimaeras. This study was compared with the combination of mesenchymal cells and cardiac progenitor cells. After four weeks of port infusion, the cardiac chimaeras treated population presented an improvement in the wall thickness. At six-week, cardiac function was improved with the cardiac chimaeras’ group and at eighteen weeks in another group. In the cardiac chimaeras’ group, infarct size, engraftment was significant (Quijada et al., 2015).

#### 9.2.8 Large animal studies

In the large animal model, a summation of the mesenchymal cells and cardiac stem cells were studied in the swine (Shake et al., 2002, Cai et al., 2016). Human mesenchymal cells and the cardiac stem cells were administered via the intramyocardial route to the immunosuppressed swine after fourteen days of myocardial infarction. The combined cells presented a two-fold reduction in the scar size, seven-fold increased engraftment, and increased left ventricular function compared to the individual cell types after four weeks. The individual cell created an important enhancement as compared with the placebo groups (Cai et al., 2016).

In another model, termed as chronic ischemic immunosuppressed swine model, mesenchymal autologous cells and cardiac stem cells were delivered three weeks post-infarction. Results include- improvement of the ejection fraction, stroke volume, cardiac output and diastolic strain as compared to the mesenchymal cell alone. Both the groups showed an increase in the scar size, and wall motion, compared with the placebo (Karantalis et al., 2015). Besides, a similar study that used mesenchymal cells and cardiac stem cells demonstrated that the combined cells produced a greater enhancement in cardiac structure and activity at least in part by increasing cell extension within the myocardium (Golpanian et al., 2016). However, the summaries of the animal and human trials on the stem cell therapies for the ailments of cardiac degenerations are mentioned in Table 5.

**Table 5:** Trials conducted on stem cell therapies to treat cardiovascular degenerations.

Nature of study	Stem cell types	Results (improvement)	Drawbacks	References
A *meta*-analysis of 33 trials	Mature/adult bone marrow-derived cells	Momentous development in the left ventricular ejection fraction was observed after myocardial infarction.	Though there was an improvement in ventricular function, it did not show an uplift in morbidity and mortality.	(Fadini et al., 2010)
REPAIR-AMI Trials	Bone marrow autologous cells	At two years, there was a better outcome and increase of the ventricular function in the patient with myocardial infarction.	The current clinical studies that demonstrate the safety and efficacy of the bone marrow-derived cells are however disheartening.	(Egeland and Brinchmann, 2007)
TIME trial	Autologous bone marrow cells are administered via intra-coronary.	BMCs were found to be safe for this high-risk group of people who kept their Left ventricular function and volume stable for two years. Nearly half of the people in the study had MVO at the start of the study, and it was found to be linked to a significant decrease in LV function recovery, an unfavorable remodeling of the LV, and more ICDs.	This study did not represent any improvement in ventricular function.	(Traverse et al., 2018)
POSEIDON STUDY	Bone marrow-derived cells were delivered via the *trans*-endocardial route in patients who were suffering from ischemic cardiomyopathy.	In comparison to scars that were not subjected to TESI, those treated with TESI showed higher SEF scores. It was shown that both sets of scars, TESI-treated and untreated, had smaller scars.	This study failed to present any improvement in the global ventricular function.	(Suncion et al., 2014)
SCIPIO Phase-1 trial	Intracoronary injection following myocardial injection with Autologous c-kit+, lineage cardio-protective cells. autologous c-kit+ CSCs	Improvement of left ventricular ejection fraction about 12.3% after 1 year when administered an intracoronary injection of the c-kit+ cells. Following a myocardial infarction. Clinical improvement is showed in the ischemic cardiomyopathy patients who were delivered intracoronary autologous cardiac stem cells. There are also some effects observed such as left ventricular improvement, a decline of the scar size and also evaluated the safety and efficacy. There observed no adverse effects after one year.	SCIPIO has limitations as a result of the small number of patients who participated in the study and the absence of the information's of individuals who received a placebo.	(Bolli et al., 2011)
CADUCEUS Phase-1 trial	Cardio-sphere derived autologous cells were administered via the intracoronary route.	A decrease in the scar size was observed and improvement in the viable tissues and contractility was observed when demonstrated by cardiac magnetic resonance after6 months. No adverse effects were observed.	There observed no important differences in left ventricular ejection fraction between the two groups.	(Makkar et al., 2012)
Randomized controlled trials consist of 22 studies; a *meta*-analysis	Bone marrow-derived mononuclear cells with acute myocardial infarction.	There observe a 2.10% increase in the left ventricular ejection fraction in the treated groups. Reduction in the infarct size.	No effects were observed in the cardiac function, infarct size. Does not enhance the cardiac function in the MRI derived parameters. No clinical outcome was observed.	(Hou et al., 2020)
PRECISE Trial	The cells were isolated from liposuction and prepared as fresh cells via endocardial injection.	Important enhancement in the left ventricular mass was observed by the MRI and wall motion score index. Decrease in the inducible ischemia in the adipose-derived group after 18 months. Preservation of the ventricular function. Myocardial perfusion and workout capacity also increased.	Because the treatment group was older, it is possiblethat they were more likely to have a poor outcome.	(Perin et al., 2014)

## 10 Conclusion and recommendation

Stem cell therapy is the novel front-line fighter in the war against cardiovascular disease. They flake exquisite research and detraction. These cell therapies are desirable options to reanimate the myocardium and flourish cardiac function after the myocardial infarction. Though stem cell therapies are one of the greatest obtainments in the treatment of heart disease, there are some irrational foundations for proving the extensive variety of stem cells and methods of isolation and production. There are some discrepancies between the methods which are inadequate to determine the attribute and strength of the stem cell preparation. There is only a small amount of study that compares the route of administration and doses of the cell therapy. There is a lack of proper data that determines the safety and efficacy of the therapy. So, it is recommended that the researcher's committee should consider a reliable method and the comparison of the cells, dose–response assessments, and route of administration in their study design. No single cell is proved to be the best approach for stem cell therapy. So, the combination of more than two cell types or combining cell and pharmaceutical approaches are recommended for the improvement of stem cell therapy. Several types of research along with clinical trials, case series, retrospective studies, cohort studies, and *meta*-analyses have been done to establish the safety and efficacy profile, mechanism of action and future aspects of stem cell therapy in cardiovascular illnesses. Although specific instances of stem therapy have proved its effectiveness in cardiovascular illnesses in certain substantial studies and have been adopted in various countries, the safety and efficacy profiles of those treatments are yet to be thoroughly established in large-scale clinical trials. Thus, establishing full safety and efficacy profiles of stem cell research is still a vital requirement and coordinated efforts throughout the world are essential to reach this objective. To investigate the possible mechanism of stem cells in improving heart function recovery, such as genetic pedigree monitoring in analyzing the destiny of transplanted cells. Furthermore, research should be concentrated on the deployment of a new generation of cardiac-function-improving techniques, such as the cardiac fibroblasts, employment of reprogramming, tissue-engineered meshes, various stem cell-derived cells, non-coding RNA exosomes and the delivery of functional genes could help us to establish the standard portfolio for the treatment of cardiac diseases by the stem cell therapy in the near future.

## Funding

The study was not financed by any organization or authority.

## Ethical Consideration

Not applicable.

## CRediT authorship contribution statement

**SM & SA:** Investigation, Formal analysis, Methodology, Resources, Software, Writing – original draft. **NUE:** Conceptualization, Methodology, Investigation, Formal analysis, Writing – original draft, Validation, Writing – review & editing. **USB:** Investigation, Data curation. **KJ:** Data curation, Formal analysis. **FA:** Supervision, Validation. **ASMMAH:** Project administration, Methodology. **AT:** Investigation, Data curation. **SR:** Data curation, Formal analysis, Project administration, Writing – original draft. **MA:** Supervision, Funding acquisition, Visualization, Project administration.

## Declaration of Competing Interest

The authors declare that they have no known competing financial interests or personal relationships that could have appeared to influence the work reported in this paper.
